# Supplementary material for: Are carbon emissions trading and green financial instruments synergistic? -Comprehensive quantitative research based on content analysis
Source: PLoS One. 2024 Mar 7;19(3):e0298601. doi: 10.1371/journal.pone.0298601 (PMC10919601; doi:10.1371/journal.pone.0298601)
Supplement: S1 Data — (ZIP) [file pone.0298601.s001.zip › Data/地级市Z_F工作报告.zip!/║ú─╧/╚2╤╟╩╨/└┤╫╘╣1⁄2╓┌║┼í╛┬φ┐╦╩2╛▌═°í┐_2014.docx]

各位代表：

现在，我代表市政府向大会报告工作，请予审议，并请市政协委员和其他列席人员提出意见。

2013年工作回顾

过去的一年，面对十分复杂的经济形势、持续加大的转型压力、日益增长的民生需求和艰巨繁重的管理任务，市政府在省委、省政府和市委的正确领导下，与全市人民一道，努力奋斗，攻坚克难，取得了新的发展成绩。全市实现生产总值373.5亿元，同比增长10.1%（按可比价格计算）。全市地方公共财政收入67.5亿元，增长16.6%。城镇居民人均可支配收入25460元，增长9.3%；农民人均纯收入9795元，增长11%。全市CPI同比上涨3.2%，价格总水平保持稳定。

一年来，我们主要做了以下工作：

一、着力扩内需，投资、消费保持较快增长。继续开展“项目建设年”工作，强化项目属地管理，加强项目督导检查，98个市重点项目去年完成投资423.6亿元，超过年度计划34.1%。全市有6个区镇超额完成重点项目投资任务。在重点项目的带动下，全市固定资产投资完成523.3亿元，增长21.6%。随着免税购物新政实施、边境游异地办证获批、邮轮游艇旅游加快发展、低空旅游投入商业运营以及三亚千古情景区开业迎宾，我市的旅游产品和游客的消费选择更加丰富，促进了消费增长。2013年全市社会消费品零售总额123.5亿元，增长17.8%。

二、加快调结构，产业发展再现积极变化。2013年全市第一、二、三产业增加值分别增长6.4％、9.2％和11.1％，三次产业结构为13.3：19.8：66.9。围绕特色优势农产品生产，扶持农民专业合作社发展，加快农业现代化步伐。补贴支持南沙生产渔船更新改造和赴南沙捕捞，海洋渔业稳步发展。加快崖州中心渔港、南山深水港等项目建设，海洋产业发展基础条件正在完善。旅游产业效益继续提升，全年接待过夜游客1228.4万人次，增长11.5%；实现旅游总收入233.3亿元，增长21.4%。房地产市场活跃，全市商品房销售面积105.1万平方米，增长70.5%。新兴高增值产业发展取得新成效，特别是金融业实现增加值36亿元，增长38.2%，拉动GDP增长2.7个百分点。阳光人寿保险业务稳定发展，国开行三亚分行、三亚农商银行、民生银行三亚分行等多家金融机构挂牌开业，三亚首个股权投资基金管理类公司获准注册。南航三亚总部基地综合楼加快建设，企业绿都等项目前期工作有序推进。创意产业园、市高新技术产业园等园区发展加快，微软、思科等国际名企落户三亚，智慧产业发展潜力增强。

三、突出“可持续”，生态文明建设继续加强。三亚湾、三亚河雨污分流项目和三亚市新城水质净化厂基本完工，生活垃圾焚烧发电、餐厨垃圾处理项目正加快推进。加强了重点污染源巡查监管。按照新标准实施空气质量监测并发布空气质量指数（AQI）。严格实施投资项目节能审查，积极推进节能示范项目建设与应用，扩大亚龙湾冰蓄冷区域供冷站服务范围，加快可再生能源建筑应用示范城市建设，全年新建民用建筑节能达标率在95%以上。省里下达的节能减排年度任务预计能够顺利完成。全面开展生态环境保护绩效审计，继续推进生态市建设。认真实施“绿化宝岛”行动，完成各类造林2.68万亩，完成退果还林4200亩。加强海洋和海岸生态环境保护，合理布局和建设公益性人工鱼礁，推动榆林红沙港联合清理整治，对六大海湾陆源污染物排海状况实施了严密监控。

四、扎实打基础，城乡建设有序推进。积极开展“科学规划年”工作，加快专项规划、城市控规和镇、村规划编制，规划覆盖面进一步扩大。完成了金鸡岭路延伸段等项目建设，凤凰机场三期改扩建、西环铁路三亚段、绕城高速公路槟榔互通连接线、农村公路通畅工程和桥梁改建维修等项目加快推进，红沙隧道前期工作有序开展。优化公交线路，开通了海棠湾、崖城公交网络。新投放了416辆出租车运力指标和200辆电召出租车。城乡电网建设进展顺利，新铺设了28公里的天然气管道。临春、榕根、东岸等片区改造项目稳步实施，海棠湾风情小镇建设、新农村综合改造建设试点有序推进。完成了病险水库除险加固、小型农田水利基础设施以及河道治理项目年度建设任务，建成了大隆灌区西干渠工程。

五、创新增活力，改革开放迈出新步伐。完成了服务业综合改革试点中期评估。继续推动三亚新大兴园林、亚龙湾云天热带天堂森林公园上市工作。完成了市中小企业信用担保中心改制。扩大农民小额贷款贴息范围和贴息额度，去年共发放50万元以下的农民小额贷款4亿元。增加了农业保险险种和财政补贴。人行农户征信系统正式上线运行。全面推进农村土地承包经营权登记，提高了农村宅基地和房屋确权登记发证率。继续推进力村全国农村集体土地产权制度改革试点。制定实施《三亚市集体建设用地开发利用审批流程（试行）》，规范了集体建设用地开发利用行为。积极服务于国家总体外交，圆满完成了博鳌亚洲论坛年会等重大国事活动的服务保障任务，促进了我市的对外交流与合作。借助“泛珠大会”等平台，引进了农业、旅游、教育、高新科技和现代服务业等领域的17个项目。去年我市实际利用外资2.4亿美元，实现进出口总值1.5亿美元。

六、全力保民生，人民群众生活不断改善。2013年拨付重点民生项目资金54亿元，同时压缩行政经费及一般性项目经费3888万元用于民生事业。全市新增城镇就业岗位3.3万个，转移农村富余劳动力就业1.5万人，城镇登记失业率为1.5%。全市城镇从业人员五项社保参保率达到98.6%，城乡居民基本医疗保险基本实现全覆盖，新型农村社会养老保险参保率超过98%，各项保障水平进一步提高。加强常年蔬菜基地建设，规范农副产品平价供应网点和直供直销门店管理，组织发放物价上涨补贴4.4亿元。全年新开工建设各类保障性住房5530套，建成6444套，完成分配2150套；完成农村危房改造1500户。加快安置区建设，吉阳福海苑安置区已建成2001套安置房，海棠湾风情小镇已建成823套安置房并实现首批搬迁入住。积极推进中小学校标准化、规范化建设，增加了230个普通高中学位。育才镇中心幼儿园已建成。扶持民办幼儿园规范发展，新增学位近4000个。基本完成了市人民医院整体改扩建二期工程，市中医院三期项目顺利推进。着力增强基层医疗卫生服务能力，安排6家城市医院对口支援8家基层医疗卫生机构。市图书馆重新开放，群众艺术馆已开建，24小时自助图书馆和农家书屋建设与管理进一步加强，群众性文化体育活动蓬勃开展。妇女、儿童、青少年、老龄、残疾人、红十字事业有了新发展，民族、宗教、侨务、口岸、地震、人防、档案、史志、人口计生等工作取得新成绩，第三次全国经济普查正在有序开展。

七、坚持强管理，社会建设水平稳步提升。进一步下放了市容、环卫等方面的城市管理权。在河西区创建数字城管示范区。依法依规拆除违法建筑124万平方米。三亚旅游系统诚信管理平台、旅游质监与投诉系统投入运行。强化旅游市场监管，扎实推进旅游旺季突出问题综合整治，赢得了今年的春节“黄金周”大考。构建多方参与的社会治理体系，提升基层治理能力，完成了全市138个村（社区）的村级组织换届选举。加强流动人口服务与管理，在河西区试行流动人口暂住证办理。继续完善社会治安防控体系，严厉打击“两抢一盗”等犯罪活动，再次荣获“长安杯”。集中力量开展全市安全生产大检查，特别是对道路交通、涉海旅游、建筑施工、消防安全、液氨使用等领域的安全隐患进行了重点排查整治。强化食品药品监管，完成了2000多家餐饮服务单位的量化分级管理评定。加强矛盾纠纷排查调处，建立群体性上访事件快速处置机制，通过各种载体拓宽了信访渠道。积极应对强台风“海燕”，安排1.46亿元用于恢复生产和灾后重建。双拥共建活动取得新成效，国防动员、民兵预备役等工作实现新进步。

八、转型增效能，政府自身建设不断强化。自觉接受人大、政协监督，一年来办复市人大代表建议115件、市政协委员提案149件，办复率100%。完成全市第三次行政审批项目清理，建立审批项目目录库，所有非涉密项目均可实现网上申报，建设项目并联审批开始试运行。2013年市政府政务服务中心各类项目平均办理时间已压缩至3.12个工作日，比法定平均办结时限少17个工作日。建立各区镇便民服务中心和26个社区居（村）委会便民服务站，方便了群众办事。进一步强化政府工作督查、督办，严格执行问责制度，认真开展绩效考评，提升了行政效能。狠抓干部作风建设，深入开展“庸懒散奢贪”问题专项整治，结合“民生体验”活动，解决了一批涉及群众切身利益的实际问题。全面推行预算项目库改革，公开了2013年市本级财政预算、部门预算以及“三公”经费预算信息，强化了财政投资评审和预算绩效评价。进一步加强政府廉政建设，加大了审计、监察工作力度。

各位代表，过去一年的成绩来之不易，这是在省委、省政府和市委的正确领导下，在市人大、市政协和社会各界的帮助支持下，全市人民共同努力的结果。在此，我谨代表市政府，向全市人民致以崇高的敬意！向人大代表、政协委员、各民主党派、人民团体和社会各界人士，向中央和省驻三亚各单位，向驻市部队、武警官兵、公安政法干警，向所有关心支持三亚发展的国内外朋友，表示衷心的感谢！

在看到成绩的同时，我们也必须正视三亚当前发展中存在的困难和问题。一是产业结构调整仍需加快。在2013年全市生产总值、全社会固定资产投资和地方税收收入中，房地产业所占比重分别达到26.6%、57.9%和62.7%，新兴高增值产业尚未形成足够强劲的增长点。二是居民增收难度较大。随着基数的不断提高，居民收入增速已出现放缓趋势，去年全市城镇居民人均可支配收入增速低于预期目标0.7个百分点。三是管理工作亟待强化。当前三亚正处于发展转型期，城市规模快速扩张，游客接待量不断增加，新问题、新矛盾非常多，各方面的管理任务十分艰巨。四是生态环保压力增大。三亚的环保基础设施还远不够完善，污水、垃圾处理和山体、森林、河岸、海岸管控与保护能力都急需增强。五是公共服务供给不足。教育、医疗、文化等方面的供需矛盾十分突出。六是政府效能仍需提高。对于这些问题，我们将高度重视，并采取有力措施，切实加快解决，让人民群众见到实实在在的成效。

2014年主要任务

2014年是全面贯彻落实十八届三中全会精神的第一年，也是完成“十二五”规划目标任务的关键一年。今年我市经济社会发展的主要预期目标是：全市生产总值增长10%左右；全社会固定资产投资增长20%左右；社会消费品零售总额增长15%左右；地方公共财政收入增长15%左右；接待过夜游客增长8%左右；城镇居民人均可支配收入增长10%左右，农民人均纯收入增长11%左右；城镇登记失业率控制在3%以内；居民消费价格涨幅控制在4%左右；完成省里下达的节能减排任务。

为了实现这些目标，我们必须按照市委六届三次全体（扩大）会议的部署，坚持稳中求进、改革创新，通过改革激发发展活力、破解发展难题，加快转变经济发展方式，稳步推进新型城镇化，着力保障和改善民生，突出抓好科学管理，推动三亚持续健康发展。今年要重点抓好六个方面的工作：

一、坚持深化改革，为三亚发展注入新的动力。把改革创新贯穿于经济社会各个领域，从制约发展最突出的问题入手，从人民群众最期盼的领域改起，重点推进九个方面的改革：

（一）加快转变政府职能。进一步清理行政审批项目、规范审批流程、严格审批时限、落实审批责任。特别是要围绕建设项目审批这一重点，落实建设项目优化审批流程试行方案，全面完成重点项目并联审批机制建设，铺开实施受理容缺制、超时默许制和联合审批缺席默认制，导入可视化监管和回溯系统，对建设项目联审情况进行定期通报。积极推进全流程网上审批平台建设，力争50%的审批事项实现全流程网上审批。建立健全三级政务服务体系，力争实现所有社区居（村）委会便民服务站全覆盖。在公共服务领域引入市场化机制，对农村公路养护等事项实行政府购买服务的模式。积极稳妥推动事业单位分类改革。

（二）启动行政区划调整工作。根据上级批复精神，以实现政府扁平化、集约化管理为目标，按照“市、区两级政府，市、区、社区居（村）委会三级管理”的基本框架，加快制定具体的调整方案和配套政策，统筹安排好行政区划勘界、机构设置、编制配备、人员调配、资产配置等事项。在确保稳定有序的前提下，要争取从下半年开始进入撤镇设区实质性操作阶段。

（三）继续推动国企国资改革。对全市85家国有关停企业的资产进行汇总分类，对符合条件的先行注销一批、处置一批、整合一批。今年要加快完成公交集团整合组建，要统筹全市城市供水管理运营。积极推进国有企业股份制改造，支持大小洞天公司上市工作。引进优质企业，通过参股、控股、资产收购等多种形式，参与市属国有企业改制重组，发展混合所有制经济。大力推动非公经济发展，引导金融机构加强对农业、服务业等领域小微企业的贷款支持。

（四）努力构建现代市场体系。推进工商注册制度便利化改革。继续开展服务业综合改革试点工作，完善现代服务业发展配套政策。深化旅游管理体制改革，按照省里的部署，逐步建立旅行社等级评定和市场退出机制，建立导游人员薪酬制度和社保制度。有序引导民间资本进入金融服务领域，支持发起设立地方性小型民营金融组织。扶持信用担保机构发展。拓宽企业融资渠道，支持符合条件的企业加快上市和“新三板”挂牌工作。进一步提升国有土地“招拍挂”出让的覆盖面，减少非公益性用地划拨，推进海域使用权市场化配置。结合城中村、城边村改造，探索推行安置拆迁费用通过招标由市场解决、安置拆迁工作由政府组织实施的模式。深入开展土地管理领域突出问题专项治理，严防非法占地、闲置土地等行为。

（五）继续深化农村改革。尽快完成农村土地承包经营权登记，根据省里的部署，探索推动经营权流转试点。积极扶持农民专业合作社建设，培育新型农业经营主体，因地制宜发展适度规模经营。完善农业企业、农民专业合作社、农户的合作与利益分配机制，促进农民增收。加快高标准基本农田建设，稳步推进城乡建设用地增减挂钩试点工作。完成农村集体土地共有宗地分割确权、登记发证，在此基础上，根据上级试点安排，严格按照规划和用途管制，探索农村集体经营性建设用地出让、租赁、入股，同时严厉查处集体土地非法流转行为。结合城中村、城边村改造，统筹解决好转移人口的技能培训、就业、社保、户籍等问题。鼓励有关区镇和企业强化组织管理、后勤服务，为农民的房产物业寻求经营创收渠道，为农民群众寻求就地就业途径。

（六）推进财税管理体制改革。根据上级部署推动“营改增”试点扩围。着力完善预算管理制度，加快项目支出标准定额体系建设，继续推动预算项目库改革，构建编制、执行、监督、绩效评价“四位一体”的预算管理体系。强化政府收支信息公开，从今年起要分别公开当年度的“三公”经费预算信息和上一年度的“三公”经费决算信息。全面开展政府性债务清理整顿，把政府性债务偿还支出统一纳入财政预算管理，完善政府性债务偿债和风险预警机制。结合撤镇设区工作，按照权责对应的原则，进一步推动财权下移。

（七）健全生态文明建设体制机制。着手编制空间管制规划，划定开发管制边界，强化山体、森林、水体、河岸带、海岸带等自然资源和生态空间管控，建立资源环境承载能力监测预警机制。在进一步完善森林生态效益补偿机制的基础上，着手制定水源保护区生态补偿工作方案，逐步健全自然资源的有偿使用和生态补偿制度。加强重点生态功能区保护，进一步完善自然保护区管理制度。在全省统一部署下，积极开展环境产权、碳排放权、排污权有偿使用和交易试点工作。

（八）推进民生领域重点改革。进一步完善和落实各类教育补助政策，制定边远地区教师奖补制度和基础教育人才支教管理规定。支持民办教育发展，加强民办学校招生管理和学籍管理。继续扶持职业教育发展，立足市场需求，加强专业建设，深化校企合作，提高中职学校办学水平。推进特殊教育发展。继续实施公立医院改革，以市中医院为试点，推进单位编制和经费拨付管理改革；以市人民医院为试点，推进医事服务费和医疗服务价格改革。着手制定鼓励社会资本兴办医疗卫生机构的实施细则。全面开展公共租赁住房和廉租住房并轨工作，探索建立多层次、全方位的住房保障体系，细化针对不同群体的保障实施标准。

（九）进一步扩大开放。争取中央和省里的支持，推动三亚国家首脑外交和休闲外交基地建设，在全力做好博鳌亚洲论坛年会相关活动服务保障的基础上，力争承办更多高层次的外事活动。依托国际旅游岛建设，通过扩大开放丰富旅游产品要素，特别是要研究邮轮公司在三亚落地的优惠政策，重点鼓励组建以三亚为注册地、与国际著名品牌邮轮公司合资的中国邮轮公司；争取扩大开放水域，延长外籍游艇访问续延时间，简化续延审批手续；充分利用低空空域开放试点的机会，争取提升空域容量，健全低空旅游准入制度和运营标准。积极参与区域发展合作，加强与泛珠、泛北部湾和港澳等地区的对接，重点引进科技含量较高、附加值高、能耗低、无污染、拉动力强的产业型项目。完善人才落户、住房、子女入学等方面的配套政策，建立健全集聚人才的体制机制。着力提升通关效率，优化人员出入境和货物进出口环境。

二、转变发展方式，努力提高经济发展质量。加快经济结构调整步伐，进一步增强消费的拉动力。通过加强生态文明建设巩固环境优势，提升三亚的可持续发展能力。

（一）继续优化产业结构。认真落实“2014年促进经济持续健康发展财政补贴”等一系列政策，加快产业转型。一是要推动特色优势产业升级。旅游方面，要大力发展邮轮、游艇、潜水、海钓等海上旅游项目，着力培育低空旅游、森林旅游、文化演艺、商务会展、婚庆蜜月、康复疗养、健康养老、乡村旅游等产品。支持三亚千古情打造文化旅游精品，加快推进凤凰岛国际邮轮港二期工程、鹿回头广场游船游艇码头、三亚国际水上飞机中心等项目，尽快开建海棠湾亚特兰蒂斯、梦幻不夜城和三亚国际教育度假区西区等项目。房地产方面，要加强产业引导和调控，建立房地产开发准入门槛和“黑名单”制度，着力提升开发品质。要鼓励优质企业开发旅游、商业、商务办公、创新创意、文体教育、康复疗养等产业型地产。滨海一线土地禁止用于普通商品房开发。农业方面，要积极发展热带特色现代农业，并促进农业观光、休闲、体验旅游发展。要抓好国家冬季瓜菜基地建设，推广农业标准化种植，提高畜牧业标准化、规模化养殖水平。依托“海南三亚国家农业科技园区”，继续推动南繁制种产业化，扩大各类生产示范基地规模，推广先进农业技术，提升科技成果转化率。加快三亚市现代农业检验检测预警防控中心建设，进一步强化农产品质量安全监管。二是要继续培育新兴高增值产业。加快阳光金融广场、三亚财经国际论坛永久会址、南航三亚总部基地综合楼等项目建设。积极推进光大财险总部落户，争取中信银行三亚分行设立。支持金融机构开发新的信贷市场，支持基金、信托、金融租赁等业态发展，鼓励保险机构创新旅游保险产品。完成西安中盈公司、海南中度实业公司设立的基金落户工作。加快“三亚云港”等信息化基础项目建设，提升城市综合信息服务能力，推动智慧城市建设和智慧产业发展。加快开展南海开发服务基地和南海旅游服务基地区域用海规划。建成崖州中心渔港基础性设施，推进南山深水港建设。鼓励大吨位渔船赴三沙海域开展常态化生产，推行生态健康养殖。探索开展海域和海岛使用权、船舶所有权、涉海技术专利权等抵押贷款业务，推动金融为海洋资源开发服务。三是要突出园区的平台作用。不断完善各园区基础设施和配套政策，促进产业集聚发展。突出临港优势，研究优化创意产业园发展定位，提升土地供应效率，狠抓项目前期工作，加快园区进驻项目建设。争取上级政策支持，推动海棠湾现代服务业产业园区早日挂牌成立。继续推动省动漫产业基地建设发展，开展3D打印机大型样机制造试验及市场推广。积极支持微软、思科、惠普等企业和赛伯乐投资基金打造专业化特色产业园。在依托园区集聚发展产业的同时，也要鼓励企业利用已有的零散项目或土地实施产业升级，鼓励三亚学院等高校开发科技创意项目。

（二）着力优化投资结构。根据城市发展和产业发展战略，通过项目立项、土地供应、规划许可、施工许可等多个环节的调控，优化投资结构。逐步建立健全投资市场监测预警机制，有效防范产能过剩。在项目推进过程中，要进一步强化跟踪服务和工作督查，严格落实“六个一”责任模式。发改等部门要加强项目指导、监督，对进展缓慢的项目要重点督办。改进征地拆迁和安置模式，鼓励项目自主配建安置区，切实落实就业、社保等后续保障措施。着力加快安置区建设，今年鹿回头整体改造安置区要完成1260套安置房建设，吉阳福海苑安置区要全面竣工验收，力求发挥示范作用。

（三）积极引进和培育消费。围绕旅游、房地产、热带农业等特色优势产品做好宣传促销。与网商平台积极开展合作，充分发挥网络营销的巨大潜力。海棠湾国际购物中心要按期正式营业，要用足用好海南免税购物政策措施，进一步提升其对三亚旅游购物的带动作用。加快推进商贸物流项目建设，完善城乡商贸服务功能。大力发展服务消费，积极培育文化、健康、信息、家居服务等消费热点，扩大本地居民消费规模。

（四）加强生态文明建设。认真开展固定资产节能登记，以建筑、交通等行业为重点强化节能管理，抓好节能日常监督和年度考核。积极开展建筑垃圾处理项目前期工作，开工建设三亚主城区雨污水收集排放系统工程，加快完善市区污水管网和三亚市新城水质净化厂配套管网，推进餐厨垃圾处理项目、崖城水质净化厂和城镇垃圾转运站等工程建设，城市中水回用工程要在年内竣工，生活垃圾焚烧发电一期项目要建成投入运行。加强重点减排单位监督管理，严厉查处非法排污行为。加强环境空气质量监测和大气污染防治。推进建设项目环境影响评价制度专项治理。继续开展“绿化宝岛”行动，今年要完成绿化造林8000亩，完成退果还林1万亩。加快丰兴隆公园项目的前期工作，白鹭公园生态恢复和景观提升工程要争取在年内开工。着力强化珊瑚礁、红树林和湿地保护。积极推进三亚湾的生态保护带建设，继续实施海域岸线重点领域突出问题专项治理，认真开展海域使用后评估。制定创建国家级海洋生态文明示范区的实施意见。

三、统筹城乡发展，稳步推进新型城镇化。立足三亚实际，结合行政区划调整，有序推进城镇化和城乡统筹发展，扩大发展空间，夯实发展基础。

（一）稳妥开展新型城镇化实践。在城市总规确定的主城区以及海棠湾、亚龙湾开发区内，以城中村、城边村综合改造和风情小镇建设为抓手，推动农民就地城镇化。今年要加快实施海棠湾风情小镇、亚龙湾玫瑰谷风情小镇等项目建设，要加大力度推动东岸、榕根、临春、海坡、月川等城中村改造，要按照既定实施方案和规划方案，先行启动凤凰镇海坡二线西瓜村和芒果村的整片改造建设。在改造和建设过程中，要注重完善市政配套设施、公共服务设施和城市功能，注重培育产业发展，为居民提供完善的后续生活保障。

（二）着力打造特色海滨城市。开展城乡风貌规划研究，保护和利用好山、河、海等自然资源，塑造三亚特色城市风貌。加强城市公共空间和景观工程设计与建设。按照国际化旅游城市的发展定位，规范建设城市地名、街名、路牌等标示标牌体系，规范管理沿街立面、临街围墙、店招牌。

（三）大力强化城乡基础建设。凤凰机场三期改扩建、西环铁路三亚段等项目要加快推进，技工学校路、春光路、绕城高速公路槟榔互通连接线等项目要建成投用，胜利路改造、绕城高速公路新联互通立交、市三环路荔枝沟段和凤凰机场段、海榆西线部分路段改造、迎宾路拓宽改造、红沙隧道、榕根桥重建、月明桥、跃进桥等工程要尽快开建，迎宾路和凤凰路地下互通项目前期工作也要加快进度。积极推进新建综合客运、货运枢纽站的相关工作，推进智能公交系统和公交首末站、换乘站、候车亭建设，健全区镇一级公交网络。通过硬件建设和科学管理，提升城区、景区停车场的服务能力。以道路网络为基础框架，加快完善城市水电、燃气、通讯、商业和社区服务等配套设施。同时，要积极推进新农村综合改造，重点实施吉阳六盘村、凤凰水蛟村、天涯文门村等片区整体改造，推进远郊村建设，并注重按照“七个一体化”的要求配置公共服务要素。要完成12项农村公路通畅工程和桥梁改建维修项目，着力抓好大隆灌区东干渠工程、田洋排沟整治、水电站改造扩容、供电安全线路改造等基础设施建设。要继续实施农村饮水安全工程，解决4.8万居民的饮水安全问题。加快推进海棠湾水系工程、宁远河、三亚东河等河流治理、病险水库除险加固以及防潮防洪工程建设，规范开展各类应急演练，进一步完善重要生活必需品应急储备体系，增强防灾救灾和应急处置能力。

四、全力改善民生，不断提升群众生活水平。坚持以人为本，继续实施重点民生项目发展规划，扎扎实实为群众办好事、办实事。

（一）千方百计促进居民增收。进一步完善就业创业政策、工资集体协商机制、最低工资制度以及劳动关系三方协商机制。鼓励农村劳动力参加职业技能培训。今年我市要新增城镇就业岗位2.5万个，实现农村富余劳动力转移就业1万人。加强农民小额贷款发放和贴息工作，扶持农民创业。强化“菜篮子”工程建设，加强物价监控，落实物价上涨补贴。抓好扶贫工作，扩大产业化扶贫覆盖面，在年内实现3500名以上的贫困人口脱贫。

（二）提高社会保障水平。进一步健全全民医保体系，保持高水平参保率，推广社保医师服务制度，扩大医疗救助范围。新型农村社会养老保险、城镇居民社会养老保险参保率要保持在95%以上，同时要按照上级部署推进二者的统筹合并工作。进一步提高城乡低保、五保户和高龄老人补助标准，抓好困难群众基本生活保障。加快推进各区镇敬老院建设。分期分批发放社保卡，逐步实现参保人员社保状况的动态化管理。

（三）完善住房保障体系。今年要新建各类保障性住房6401套，建成2436套，完成农村危房改造1800户。保障房选址要避开地段偏远、基础设施不完善的区域。对企业参与配建保障房要进行合理控制和规范管理。要把好保障房审核分配关，健全退出机制，确保公开、公平、公正分配和合理使用。

（四）推进社会事业发展。加快全市中小学校规范化建设、农村寄宿制学校改扩建和区镇中心幼儿园建设，抓好三亚湾学校征地拆迁工作。继续实施农村和城乡结合部公办中小学校营养工程。继续推进市中医院三期项目，加快市妇幼保健院、市疾病预防控制中心整体搬迁建设，加紧筹建市第二人民医院。着力改善基层医疗机构就医条件，鼓励城乡医院之间探索建立更加紧密的业务指导和双向转诊关系。加快区域医疗信息化建设，推动全市医疗资源共享。力争完成全国慢性病综合防控示范区创建工作。加强公墓建设与管理，继续推行殡葬改革，落实惠民殡葬补贴政策。继续实施文化惠民工程，积极推进群众艺术馆和农家书屋等设施建设，增加广场、公园等公共场所和社区的体育健身器材配备。广泛开展群众性文化活动、全民健身运动，抓好文化下乡工作，丰富群众文化体育生活。加强非物质文化遗产保护，启动落笔洞人类古遗址保护与开发利用工程。加快发展妇女、儿童、青少年、老龄、残疾人以及红十字事业，继续做好民族、宗教、侨务、地震、人防、统计、档案、史志、人口计生等工作。

五、强化科学管理，促进三亚发展提质增效。针对当前管理工作中的困难和问题，以旅游管理、城市管理和社会治理为重点，努力提升我市的科学化管理水平。

（一）加强旅游市场管理。继续完善旅游公共服务体系，加快实施智慧旅游管理，健全市场主体诚信管理体系，强化行业协会的管理和自律作用。依据《旅游法》，进一步强化市场监管，着力规范价格管理、计量管理、服务的标准化管理，强化旅游合同和旅游行程管理，围绕“一日游”、涉海游、海鲜排档、水果摊位等重点开展市场专项整治，坚决打击欺客宰客等违法违规经营行为，推动有序竞争，提升服务品质，打造具有三亚特色的涉海旅游、海鲜美食、热带水果和旅游商品品牌。

（二）提升城市管理水平。一是要完善管理机制和手段。结合撤镇设区工作，下放管理权，大力强化属地的管理作用和力量。进一步优化城市管理执法协作机制。扩大全民参与城市管理活动的效果。加快完善数字城管系统并尽快全面投入使用。二是要抓好管理队伍建设。切实加强相关执法人员的培训和管理，提高执法水平，坚持规范执法、文明执法。三是要突出重点问题专项整治。继续强化部门协作，坚决打击违法建筑，特别是要集中力量对凤凰、河东、吉阳等违建严重区域进行整治。一方面要按照合法、规范的程序，组织拆除典型违法建筑；另一方面要落实属地责任，加大日常巡查力度，及时发现和制止新增违法建筑；同时还要提高规划管控水平，实现规划报建程序便利化，引导居民依法报建。加强交通分流疏导，特别是要针对主要道路、重点部位和拥堵时点加强引导。扎实开展道路交通专项整治，加大路面管控力度，规范停车秩序和道路交通行为。努力营造整洁优美的市容环境，加强重点区域和时段的环卫巡查，及时清扫保洁，大力查处各种破坏市容环境的乱象，积极开展乡村环境综合整治工作。

（三）强化社会治理与服务。发挥政府主导作用，支持社会组织和人民团体广泛参与，加强基层社区建设，大力推行网格化社会治理与服务。进一步加强精神文明建设，切实抓好“六五”普法和法律援助、法律服务、仲裁调解等工作。根据省里的要求，合理确定我市落户条件。在全市推行流动人口居住证服务管理制度，构建属地化的流动人口管理体系。依托“110”警务岗亭、视频监控系统等载体，提升社会治安巡逻防控的覆盖面和工作水平，加快构建立体化治安防控体系。持续开展禁赌、禁毒，打击“两抢一盗”犯罪等行动。严格落实安全生产责任制，不定期开展安全生产检查，排查隐患，严防重特大事故。加强食品、药品、保健食品、化妆品和医疗器械监管。畅通信访渠道，加强信访督办，提高矛盾纠纷排查调处效率。进一步加强国防动员和民兵预备役工作，大力支持国防建设，广泛开展双拥共建活动。

六、抓好自身建设，打造人民群众满意的政府。顺应改革发展的新要求和人民群众的新期待，着力打造法治政府、务实政府和廉洁政府，切实增强发展能力和服务能力。

（一）加强法治建设。严格按照法定权限和程序行使权力、履行职责，坚决执行市人大及其常委会的决议、决定，自觉接受人大法律监督、工作监督和政协民主监督，自觉向人大报告、与政协协商政府重大事项。进一步健全并严格落实科学民主决策机制，完善重大行政决策程序，完善听证、公示和专家咨询论证等制度，提高公众参与度。特别是涉及重大公共利益、人民群众切身利益的决策事项，必须依法向社会公开征求意见或举行听证会。进一步完善行政执法体制，规范执法程序，落实执法责任，细化、量化自由裁量权。加强行政复议工作。

（二）加强作风建设。认真开展党的群众路线教育实践活动，在全市兴起密切联系群众和求真务实之风，坚决抵制“四风”。大力精简会议和文件，强化政府工作督查，特别是要抓好重大决策和重点工作跟踪督办，进一步完善政府绩效考评工作，落实问责制度，整治“庸懒散奢贪”现象。常态化开展“民生体验”活动，推动领导干部深入基层，到群众意见多的地方去，了解群众疾苦和诉求，自觉创新工作方法，真正解决群众困难。严格落实厉行节约、反对浪费的有关规定，把更多的财力、物力用在群众最迫切需要解决的问题上。

（三）加强廉政建设。坚持惩防并举、注重预防，深入贯彻落实党风廉政建设责任制，严格落实重要岗位和重点领域权力运行制约制度，加强廉政风险防控工作。继续深化政务信息公开，做到行政程序向社会公开，行政行为接受社会监督，确保权力规范运行、阳光运行。强化政府部门履职情况监督，加强行政监察和审计监督。严肃查处重点领域和关键环节的腐败案件，切实解决发生在群众身边的腐败问题。

各位代表，全面深化改革的新征程已经开启。让我们更加紧密地团结在以习近平同志为核心的党中央周围，在省委、省政府和市委的坚强领导下，团结依靠全市人民，不断开拓进取，为谱写三亚改革发展的新篇章而努力奋斗！
